# The healing power of sensory neurons: New horizons for diabetic and neuropathic tissue repair

**DOI:** 10.1002/ctm2.1813

**Published:** 2024-08-21

**Authors:** Yen‐Zhen Lu, Sanjay Ramakrishnan, Mikaël M. Martino

**Affiliations:** ^1^ European Molecular Biology Laboratory Australia, Australian Regenerative Medicine Institute Monash University Melbourne Australia; ^2^ Institute for Respiratory Health University of Western Australia Perth Australia; ^3^ Laboratory of Host Defense, World Premier Institute Immunology Frontier Research Center Osaka University Osaka Japan; ^4^ Victorian Heart Institute Monash University Melbourne Australia

**Keywords:** chronic wounds, diabetes, immune system, sensory neurons

## 1 INTRODUCTION

Tissue repair and regeneration after injury is a highly complex process involving the coordination of multiple biological systems. Therefore, successful regenerative medicine strategies should harness the key mechanisms that control the tissue healing process, particularly when these mechanisms are disrupted by pathological conditions that impede normal healing. Nociceptive sensory neurons, or nociceptors, are specialized primary sensory neurons with nerve endings in tissues such as skin, muscles, and joints that detect and respond to noxious stimuli, including inflammatory mediators.^1^ Although nociceptors have been shown to have pro‐inflammatory activities in some contexts, they generally mediate anti‐inflammatory processes^1^, ^2^ and their activation has been shown to be involved in skin wound healing.^3^, ^4^

## 2 A NEURO‐IMMUNE‐REGENERATIVE‐AXIS DRIVEN BY CALCITONIN GENE‐RELATED PEPTIDE SENSORY NEURONS

**FIGURE 1:**
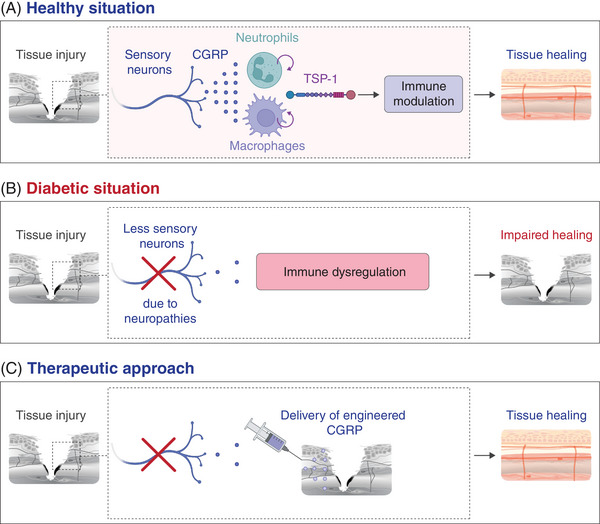
(A) Nociceptive sensory neuron endings grow into injured tissue following acute injury and release the neuropeptide calcitonin gene‐related peptide (CGRP). CGRP signalling in neutrophils and macrophages triggers the release of TSP‐1, which modulates these immune cells in an autocrine/paracrine manner to create a pro‐healing environment. (B) In the diabetic condition, there are fewer CGRP sensory neurons in tissues, and thus less CGRP is released at the site of injury, contributing to impaired tissue healing. (C) As a therapeutic approach, an engineered form of CGRP with improved pharmacokinetics is delivered into the injured tissue to accelerate the healing process.

Lu et al. investigated the role of peptidergic nociceptive sensory neurons in tissue repair and regeneration following acute injury in mice, exploring whether neuro‐immune interactions could be harnessed to promote tissue healing.^1^ They found that nociceptors extend their nerve endings into injured skin and muscle after acute injury and release calcitonin gene‐related peptide (CGRP). CGRP from sensory neurons modulates neutrophils and monocytes/macrophages—the majority of immune cells accumulating in injured tissues—to create an anti‐inflammatory and pro‐healing environment. Mechanistically, the immunomodulatory and pro‐healing effects of CGRP were mediated by the release of the extracellular matrix protein thrombospondin‐1 (TSP‐1) from neutrophils and macrophages, although CGRP may also exert direct effects. TSP‐1 was shown to act in an autocrine/paracrine manner, promoting neutrophil efferocytosis by macrophages (clearance by engulfment) and polarizing macrophages to a pro‐repair phenotype. Both processes are crucial to proper and timely tissue healing. In addition, TSP‐1 was found to accelerate the death of neutrophil and pro‐inflammatory macrophage , which could further contribute to reducing inflammation (Figure 1A). Despite these findings, a few questions still remain. For example, the precise mechanisms by which nociceptors are activated after acute injury and how nerve endings grow into injured tissue are not fully understood. Additionally, there may be critical feedback interactions between immune cells and nociceptors both at the injury site and in the dorsal root ganglia, where the cell bodies of sensory neurons are located. Furthermore, the neuro‐immune‐regenerative axis may also be significant in other tissues or contexts that involve similar mechanisms to tissue healing, such as cancer^2^ and fibrotic tissue formation, both of which rely heavily on immunoregulation.

## 3 CLINICAL IMPLICATIONS AND TRANSLATION

The discovery of a neuro‐immune‐regenerative axis mediated by CGRP is particularly relevant for patients with diabetes, as more than half of diabetic patients develop peripheral neuropathy, characterized by dysfunctional peripheral nerves and a decrease in intraepidermal nerve fibres.^5^ The resulting decrease in CGRP levels associated with neuropathies may disrupt neuro‐immune interactions crucial for tissue healing, which could contribute to the chronic and poorly healing wounds often seen in diabetic patients. Indeed, these wounds, along with the development of muscle atrophy, are among the most prevalent and severe complications of diabetes (Figure 1B). Therefore, Lu et al. tested whether localized delivery of CGRP could be used as a therapeutic strategy to accelerate skin wound healing and muscle regeneration in a mouse model of type 2 diabetes which displays peripheral neuropathies and mimics aspects of human chronic wounds (the *Lepr^db/db^* mouse). Since CGRP is a small peptide with a short half‐life, its activity following delivery is short‐lived and presents a challenge for achieving sustained effects when delivered locally into tissue. In addition, delivering CGRP without a controlled release delivery system could potentially lead to local and systemic side effects. For example, CGRP may increase nociceptor sensitivity and heighten pain responses. Additionally, CGRP is a key component in the aetiology of migraines. Furthermore, CGRP is a potent vasodilator that can also directly increase heart rate and contractility.^6^ To circumvent these potential issues, CGRP was engineered to bear an N‐terminal extracellular matrix‐binding sequence and a protease‐sensitive linker, allowing its localization and retention at the site of delivery and its gradual release to sustain activity. The delivery of engineered CGRP improved diabetic skin wound repair and muscle regeneration by reducing pro‐inflammatory neutrophil and macrophage accumulation and decreasing pro‐inflammatory cytokine production (Figure 1C). This suggests that a therapy consisting of delivering CGRP or CGRP analogues with improved stability and pharmacokinetics could be effective in promoting tissue healing in diabetic patients as well as in various conditions where immune responses are dysfunctional and the peripheral nervous system is compromised. Such conditions include the elderly population, patients suffering from burn injuries, and those with ocular neuropathy. For instance, topical application of native CGRP has been shown to facilitate corneal mechanical wound healing in mice by promoting epithelial migration and modulating immune cells.^7^

The critical role of CGRP in tissue healing raises an important question regarding the use of CGRP inhibitors in the clinic. Indeed, monoclonal antibodies against CGRP, such as erenumab, galcanezumab, fremanezumab and eptinezumab, as well as gepants,^8^ which act as CGRP receptor antagonists, are approved for use in migraine treatment and prevention. While CGRP inhibition has not been linked with major adverse events yet, case studies have reported that the use of CGRP monoclonal antibodies to treat patients with migraine was associated with worsened systemic inflammatory pathology and severely impaired wound healing in some patients.^9^, ^10^ These findings indicate that while CGRP inhibitors are effective for migraine management, there is a need for caution, particularly in patients with a tendency towards impaired wound healing or those undergoing surgical procedures. Further clinical monitoring is required to better understand and manage these potential side effects related to inflammation and tissue healing.

## 4 CONCLUSION

Although important questions still need to be addressed with further research, Lu et al. have unveiled a critical neuro‐immune‐regenerative axis that could be transformative for regenerative medicine. This discovery can be leveraged to design novel regenerative therapies for patients suffering from diabetes and neuropathies, potentially improving healing outcomes for a variety of conditions and enhancing overall quality of life.

## AUTHOR CONTRIBUTIONS

Yen‐Zhen Lu, Sanjay Ramakrishna and Mikaël M. Martino wrote the manuscript. Mikaël M. Martino prepared the figure.

## CONFLICT OF INTEREST STATEMENT

Monash University has filed for patent protection on the molecular design described herein, and Yen‐Zhen Lu and Mikaël M. Martino are named as inventors. Sanjay Ramakrishna declares no conflict of interest.

## ETHICS STATEMENT

Not applicable.
